# Editorial

**Published:** 1860-07

**Authors:** 


### Editorial.

### THE DENTAL MEETINGS.

As the time for these meetings draws near, the interest of the profession in regard to them increases. There seems to be a very general desire to be present at them. This is gratifying. Those who know most about the advantages to be gained by associated effort appreciate these meetings most highly, and make the greatest sacrifice to avail themselves of the opportunities thus presented.

There is an occasional exception, as in the case of those who go upon the *rule* or *ruin* principle ; but fortunately the number of these is so small, that they can not change the course of things very materially. It is important that the profession generally attend these meetings.

There is a general impression that the meeting at Washington will be exclusive in its character, admitting none but delegates from prop·rly constituted Societies. The draft of a constitution, which has been prepared, and will be presented for amendment and adoption, provides foı three classes of members, viz : delegates, members by invitation, and permanent members. Judging from this, it is probable that members will be made in more ways than one ; and in any event, all who go will be admitted to the meetings, and possibly to participate in the discussions.

We hope and anticipate that this Society will be established upon a permanent basis, one that shall grow stronger and stronger as it grows older. T.

### GOLD FOIL.

We have been using for a short time "Excelsior" Gold Foil, non-adhesive, manufactured by J. T. Toland, of this city. The sample we have used we have never seen excelled for soft foil. It seems to be perfectly pure, is soft and tenacious. With the perfection to which the various preparations of gold are brought, there is hardly any excuse for poor fillings. Yet we find much cheap foil in market, and many buy it because it is cheap, at the store, but forget that it is very dear at the office. Try T.'s "Excelsior."

T.

### THE MEETING AT WASHINGTON.

We have just seen a letter from Dr. McFarlan, of Washington City, to Prof. J. Taylor, of this city, in which it is stated that a very fine Hall in the Smithsonian Institute has been tendered to the Society for its meetings, and the committee has accepted the kind and generous offer. The meetings will, therefore, be held in the Smithsonian Institute, on Tuesday, the 31st of July, at 12 M.

T.

### BIBLIOGRAPHICAL.

"Dental Anomalies, and their influence upon the production of Diseases of the Maxillary Bones. By A. H. Forget, M. D., C. L. D., etc."

This is a neat little work, issued by Jones & White, published within the last year in the Cosmos, but now in book form, which is by far the most convenient. It consists of two chapters. The first treats of "Anomalies of nutrition and development." Cases are given, the first of which is very minute in all its parts; the *history of the disease,* the *condition* of the patient, the *diagnosis* of the disease, the *operation,* the *method of its performance,* with *remarks on the same,* the *consequences of the operation,* the *final results of the operation, with reflections.*

Other cases are also given, which, taken together and closely examined, afford a large amount of information.

Chapter second treats of Anomalies of position of the teeth, their pathological consequences. This part is also illustrated with cases of much interest, which it is necessary for the reader to examine in connection with the plates in the work, in order to fully comprehend them. The plates are very fine, and illustrate the subjects very perfectly. This addition to dental literature should have a place in every dentist's library. It is for sale at the dental depots. Bound in paper at 50 cts., and in cloth, at 75 cts. T.

"A Treatise on Medical Electricity, Theoretical and Practical. By J. Althaus, M. D.

This is a work just published by Lindsay & Blakiston. From the cursory examination which we have been able to give it, we hesitate not to pronounce it one of great value. It goes very thoroughly over the ground of medical electricity, treating fully of its application to and influence upon the numerous conditions for the treatment of which it has been employed.

The manner of using it in its various forms is very fully described, and the results of its application in various affections.

The work, of course, is not wholly original, and it would be less valuable if it was. Hitherto the mass of information upon this subject lay scattered all over the field of medical literature, and it was a very important work to gather it up, arrange and systematize in such a manner as shall make it most available in practice.

This is a subject of much importance to the dentist. It is but a little while since electricity was called to the aid of the dentist in the extraction of teeth; and though its use for this purpose has been in a great measure abandoned, yet we think the difficulty rested in the ignorance, awkwardness or impatience of those attempting to use it, rather than from a want of efficiency, when properly applied, of electricity itself. But leaving this altogether out of view, we have the fullest evidence of its value and efficiency, in many affections, especially those of a nervous character, with which the dentist comes in very close and frequent contact.

The work demonstrates that electricity is valuable in diagnosis, as well as treatment. Every dentist should study this subject thoroughly, and to no better source for aid can we direct him than to this work. T.

Anatomy of the Fifth Pair of Nerves.

Jones & White have just issued a very beautiful and perfect plate, exhibiting the anatomy of the fifth pair of nerves. The plate is about 18 by 24 inches, and is a very fine thing. With it is a concise description of the plate in pamphlet form. These will enable any one to study the fifth pair of nerves with great facility. The author is Henry A. Daniels, M. D., and we can not do less than congratulate him for the very perfect manner in which his work is accomplished. It is for sale at the Dental Depots. Price $2.50. When in a neat frame, it is quite ornamental. It should be in the office of every dentist. T.

---

### THE SOUTHERN DENTAL EXAMINER.

This is a new monthly dental journal, published at Atlanta, Ga., edited by J. P. H. Brown; with Geo. S. Fouke, of Md., corresponding editor. It is published at $1 per annum. We hail the advent of the Examiner as an omen of good to the profession, and especially to the profession of the South. The men who have it in charge are able to make it an interesting and valuable acquisition to dental literature. The first and second No's are before us, and give evidence of the ability of the editors.

---

### TEETH.

We have recently been using teeth from the manufactory of Dr. Porter, of Bridgeport, Ct. The Doctor has deservedly a high reputation in the manufacture of teeth, but not more than he merits.

These teeth are very firm, they are good in every point, particularly in shade and form, they are excellent. We have used them only for rubber work, but we doubt not that they would be just as good for any other kind of work. We should be glad to see more of them in market. We are confident they would become general favorites. T,

---

### A TRIP TO BALTIMORE AND WASHINGTON.

A short time ago, we had occasion to pass over the Baltimore and Ohio Railroad to Washington City and Baltimore. We had passed over the road several times before, not within the last two years, however. But never till now did we have any proper conception of the road or the country through which it passes.

The road is one of the best in the United States, notwithstanding the natural difficulties that were in the way of its construction. These to an ordinary mind would originally have seemed insurmountable, but they have all been overcome.

It is a smooth, solid road, over which the cars glide so smoothly that one scarcely knows he moves. The structure of the road seems to be perfect in all its parts, notwithstanding the ruggedness of the country through which it passes ; mountains are bored through, and the dashing train drives on through its rock-bound course with perfect security. Next comes the almost bottomless chasm, over which the train leaps, on the light and airy structure prepared for its way, with the same assurance of safety as though its track was upon a broad bed of granite.

We never traveled over a road on which we felt a greater security, and this seemed to be the feeling of all the passengers. There was an entire absence of that care and anxiety that is so frequently observed in travelers passing over roads that have not a good reputation for security.

The very perfect condition of this road is due to its superior management. The government of the road is evidently in good hands ; all its running arrangements seem to be as perfect as it is possible to make them ; the utmost care is exercised in regard to accidents ; the road is lined with watchmen, and anything out of the usual course, occurring upon the road, is always communicated to approaching trains in time to prevent accident.

The scenery along this route is worth a journey across the continent to see. The magnificent views that roll in rapid succession before the eyes of the astonished beholder, are indescribable—no tongue can tell or pen describe their beauty and grandeur. The towering mountain, with its graceful sweeps and bold curves, stands with its foot in the deep chasm, the bottom of which can only here and there be seen.

The cars roll along the mountain side, far, far above the tops of the highest trees below, and mountain tops reaching far above. In order to attain any kind of an accurate idea of the country, it must be seen. Every one should visit this region ; and we think seeing it once would increase the desire to see it again. T.

